# Primary biliary cholangitis with contemporary presence of anti-mithocondrial and anti-rods and rings autoantibodies: literature first case

**Published:** 2019

**Authors:** Roberto Assandri

**Affiliations:** *Clinical Investigation Laboratory, ASST Crema, Largo Ugo Dossena- Crema, Cremona, Italy *

**Keywords:** Primary biliary cholangitis, The rods and rings (R&R) Autoantibodies, Anti-mitochondrial autoantibodies

## Abstract

Primary biliary cholangitis, previously known as primary biliary cirrhosis (PBC), is the most common autoimmune diseases of the liver (ALD). Patients with PBC may present with typical biliary pattern symptoms. Also the presence of anti-mitochondrial autoantibodies (AMAs) is the laboratory hallmark of PBC, which molecular target antigens are members of 2-oxoacid dehydrogenase complex of enzymes.

In recent years, autoantibodies (Aab) targeting subcellular structures described as the rods and rings (R&R) pattern in HEp-2 ANA have been presented as a unique and particular case of Aab generation. These R&R structures are composed of inosine monophosphate dehydrogenase type 2 (IMPDH2), and their formation can be induced *in vitro *by several small-molecule inhibitors.

Aab targeting these relatively unknown structures have been almost exclusively observed in hepatitis C virus (HCV) patients who have undergone treatment with pegylated interferon-a/ribavirin (IFN/RBV) therapy. Literature showed that anti-RR Aab have not been found in no treatment-naïve HCV patients or in patients from any other disease.

Now we present and characterized a case patient with contemporary presence of AMAs and R&R Aab in PBC, without any laboratory evidence of HCV and/or other hepatic virus infection. For our knowledge, this is the first case described in the Literature.

R&R Aab in patients without any clinical/laboratory signs or symptoms of Hepatitis virus and without pharmacological therapy open the window to the alternative scenario: the association of these Aab to ALD. Our work demonstrated that R&R Aab can be present in PBC case. The interesting idea suggested that R&R Aab may be present also in AMA-negative PBC and they can considered a new possible diagnostic tool in this specific clinical condition.

Other study and cases are needed but the presence of R&R Aab linked with AMAs and PBC may be explained by alterations in immune regulation caused by autoimmunity in a particular genetic background.

## Introduction

 Primary biliary cholangitis, previously known as primary biliary cirrhosis (PBC), is an autoimmune cholestatic disease of unknown etiology with a varying geographical incidence and prevalence ranges between 1.91 and 40.2 per 100,000 population (1). 

Following the American Association for the Study of Liver Diseases Guideline, PCB diagnosis is certain when two of these three criteria are present: 1) biochemical evidence of cholestasis based mainly on alkaline phosphatase (ALP) elevation; 2) the presence of anti-mitochondrial antibodies (AMAs) and 3) histological evidence of intrahepatic destructive cholangitis of interlobular bile ducts (2). 

Immunological biomarkers were fundamental in the diagnosis of PBC. Even today, the presence of AMAs is one the three major diagnostic criteria (2). 

Found in more than 90% of patients with PBC, AMAs are not related to disease progression (3) and their levels do not significantly change over time (4).

In recent years, autoantibodies (Aab) targeting subcellular structures described as the rods and rings (R&R) pattern in HEp-2, have been presented as a unique and particular case of Aab generation. These R&R structures are composed of inosine monophosphate dehydrogenase type 2 (IMPDH2), and their formation can be induced *in vitro *by several small-molecule inhibitors (5).

Aab targeting these structures have been almost exclusively observed in hepatitis C virus (HCV) patients who have undergone treatment with pegylated interferon-a/ribavirin (IFN/RBV) therapy (6). 

The prolonged exposure to interferon-α and ribavirin appeared to increase the prevalence of this Aab (7). In rare cases anti-R&R Aab can be detected in non-hepatitis patients (5)

It is more significant to note that anti-R&R Aab may also be a component of autoimmune responses (8). However, literature do not report any works with clear association with R&R Aab and AD, particularly ALD.

For these reasons I present and characterized a case patient with contemporary presence of AMAs and R&R Aab in PBC, without any laboratory evidence of HCV and/or other hepatic virus infection. For our knowledge this is the first case described in the literature. 

## Case Report

**Clinical evidences**

A 46-year-old white woman was referred to our physicians for symptom related to biliary tract injury (pruritus, abdominal pain, and fatigue), without jaundice. With a history of smoking, no other clinical information has been noted to this patient.

Baseline laboratory data obtained from blood sampling revealed alkaline phosphatase (ALP) elevation together with g-glutamyl transferase (gGT), aspartate aminotransferase (AST) and alanine aminotransferase (ALT). Bilirubin level was normal (Table 1).

The differential diagnosis will go through the evidence of specific etiological agent that cause disease. For this reason, we take into consideration three clinical hypothesis: viral, pharmacological and autoimmune aetiology. Viral serologies, summarized in table 1, was considered exhaustive to exclude viral aetiology. 

Many drugs can cause cholestasis or cholestatic hepatitis, including sulfamethoxazole/ trimethoprim, amoxicillin/clavulanate, antiepileptic medication, and antituberculosis drugs. Histologic findings are variable but usually include mild portal inflammation, ductular reaction, and cholestasis. Clinical history is important, because a patient’s medication list should be reviewed for potentially offending substances, and the length of symptoms is important as well. 

Patient not referred the use of these and others pharmacological therapy, included “homemade” herbal substances. 

**Table 1:** Laboratory features of patient

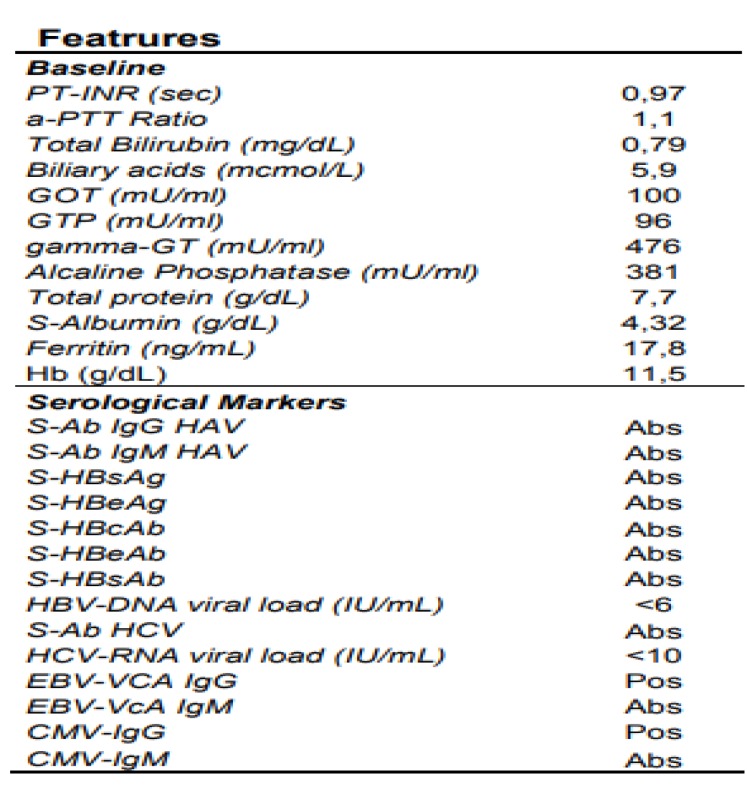

Hb= Haemoglobin; Ab= Antibodies; EBV= Epstein Barr Virus; CMV= Citomegalovirus; Abs= Absent; Pos= positive

**Immunological evidences**

**Figure 1:**
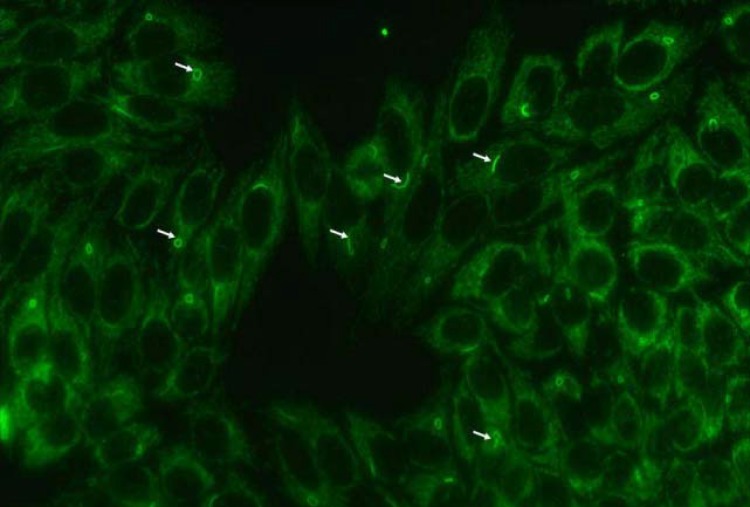
Distinct Cytoplasmic patterns identified in Hep-2 ANA IIF (Euroimmun)

A two distinct cytoplasmic pattern were identified in a routine ANA test using HEp-2 cell slides from Euroimmun, at end dilution of 1:1280 (Figure 1, table 2).

**Figure 2:**
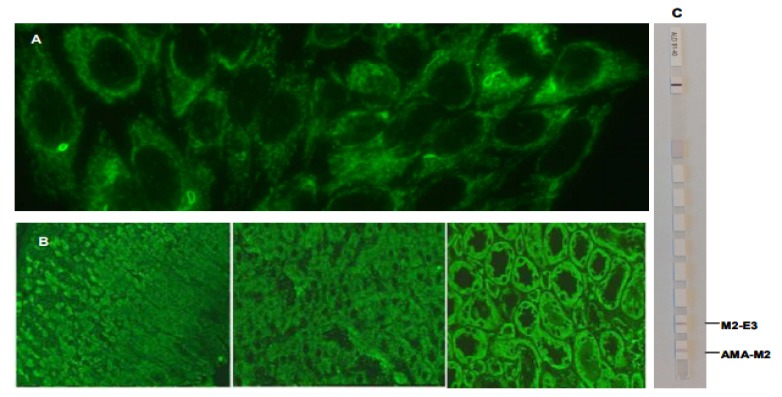
Mitochondrial like cytoplasmic pattern: Panel A: Hep-2 IIF pattern; Panel B: IIF on commercial rat liver, kidney and stomach; Panel C: Line immunoassay ALD profile.

**Table 2:** Immunological features of patient

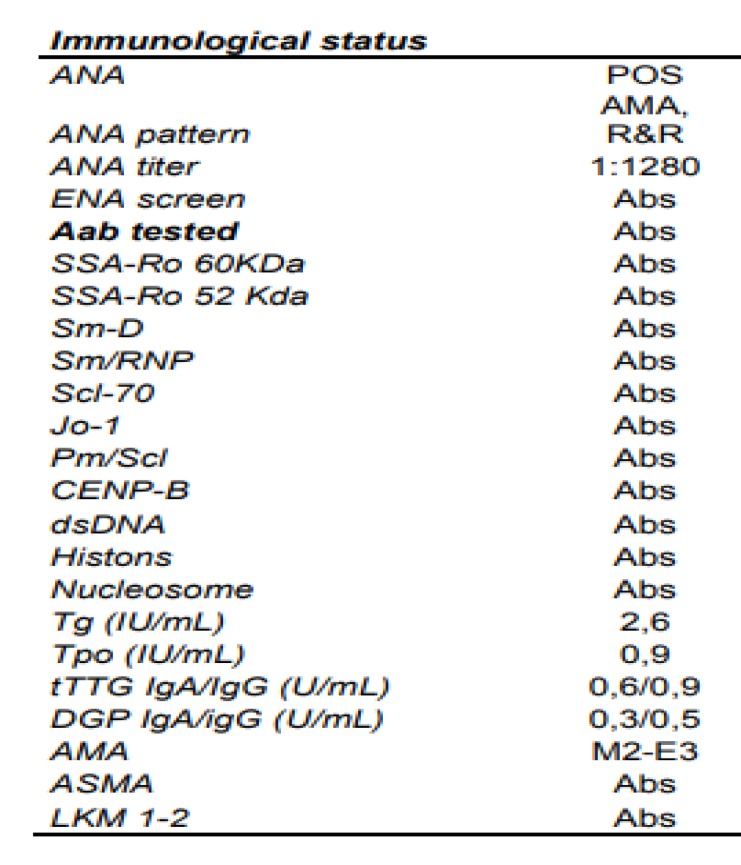

ANA=Antinuclear antibodies in IIF; ENA= Extraible nuclear antigens; Tg= Tyreoglobuli; Tpo= Tyreoperossidase; tTTG= tissue transglutaminase; DGP= deamidate Gliadins peptides; Abs= Absent

**Mitochondrial-like cytoplasmic pattern**

IIF- pattern were characterized by the presence of larger irregular granules extending from the nucleus throughout the cytoplasm in a reticular network. Cytoplasm of dividing cells was strongly positive (Figure 2, Panel A). IIF on a commercial rat liver, kidney and stomach tissue with the use of polyclonal IgG antibodies confirmed the presence of AMAs, with a characteristic staining pattern: granular diffuse cytoplasmic staining of the Kupffer cells and hepatocytes, of the renal tubules (strongest staining is noted in distal which is mitochondria-rich) and parietal gastric cells (Figure 2, panel B). Line immunoassay Euroline profile autoimmune liver diseases (IgG; LIA, Euroimmun Lübeck**) **revealed antibodies against AMA-M2. M2-E3 (BPO) but not against Sp100, PML, gp210, LKM-1, LC-1 and SLA/LP (Figure 2, panel C).

**Figure 3:**
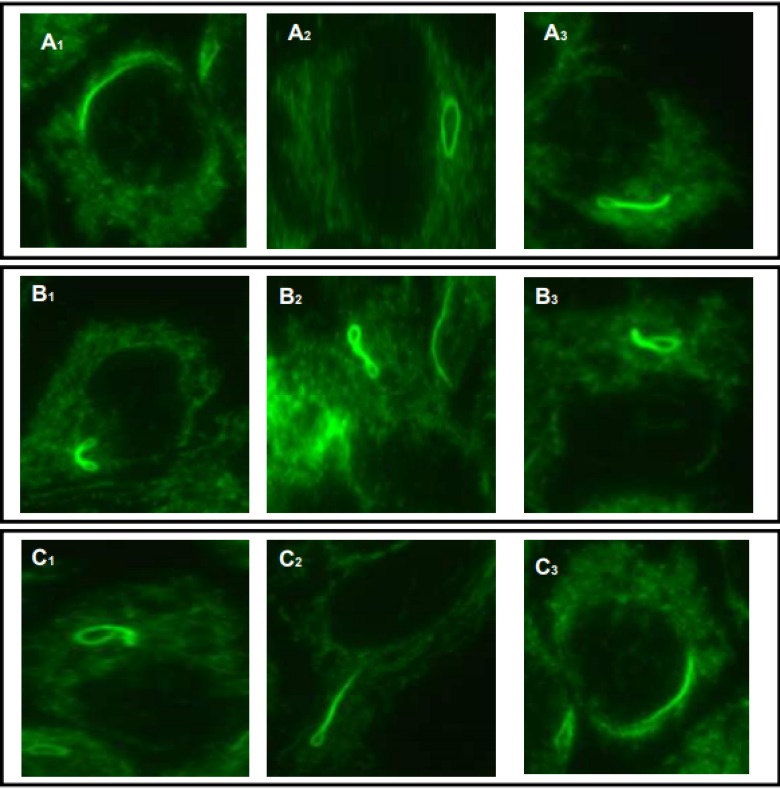
Examples of various intermediate R&R structures in HEp-2 cells stained with patient serum (FICH-green): Curved rod adjacent to the nuclear envelope (A1; C3); elongated ring (A2); elongated, twisted ring (C2); rod with a pin-loop (A3; B3;C1); Figure ‘‘8’’ structure (B1;B2)

**Rods and rings cytoplasmic pattern**

The structures recognized by patient serum were distinct cytoplasmic rods and rings (figure 1 and 3). We observed one to two rods and/or rings per cell including some intermediate structures such as a figure ‘‘8’’ (Figure 3, B1; B2), elongated rings (Figure 3, A2 ), twisted rings (Figure 3, C2), rods with pin loops (Figure 3). Some rods often align adjacent to the nucleus or perpendicular to the nucleus, and rings may be found in the cytoplasm (Figure 3, A3; B3; C1).

**Histological evidences**

The typical findings of PBC on hematoxylin-eosin stain were appeared, as described below: moderate lymphoplasmacytic portal inflammation with moderate interface hepatitis and bile ductular reaction along the periphery of the portal tracts, typical for early disease stages (Figure 4 panel A and B). 

**Diagnosis**

Following the American Association for the Study of Liver Diseases Guideline, PCB diagnosis was made:

1) Biochemical evidence of cholestasis based on alkaline phosphatase (ALP) elevation; 

2) The presence of anti-mitochondrial antibodies (AMAs);

3) Histological evidence of intrahepatic destructive cholangitis of interlobular bile ducts. 

## Discussion

PBC is the result of complex interactions between genetic predisposition and environmental triggers. 

Geographical clustering of cases has been described around areas of toxic waste disposal and in areas strongly associated with increased pollution, cigarette smoking, and toxin exposure (9). PBC has also been associated with infectious agents, hair dyes, nail polish, and cigarette smoking, although a causative role has not been established (9).

The prevalence of PBC is higher in families with an affected member, and 1-2% of children of patients develop the disease (10). Alkaline phosphatase increased levels upper limit of normal sustained by gGT increased level, accompanied by the demonstration of AMAs and relative typical histological pattern defined the diagnosis in our patient. 

AMA is a crucial hallmark for the diagnosis of PBC as their positivity, along with elevated cholestasis markers, allows a definitive diagnosis. AMA is present in about 95% of patients with PBC (11) AMAs target a family of mitochondrial enzymes, the 2-oxo-acid dehydrogenase complexes, which include pyruvate dehydrogenase (PDC-E2), branched chain 2-oxo-acid dehydrogenase (BCOADC-E2), and 2-oxo-glutaric acid dehydrogenase (OADC-E2) PBC (11). AMA titre of 1:40 or more is considered as a positive result. Magnitude of AMAs does not correlate with severity of disease and does not use for patient follow up. AMA-M2 is the subtype mostly used as a routine diagnostic marker of PBC, with a sensitivity of 55.7–79.7% and specificity of 91.7–95.4% (12). 

The typical findings of PBC on hematoxylin-eosin stain include mild to moderate lymphoplasmacytic portal inflammation with variable interface hepatitis, distorted interlobular bile ducts involved by lymphocytes and bile ductal reaction along the periphery of the portal tracts in early disease stages (11).

The novelty of this paper was the evidence of contemporary presence of R&R and AMA-M2 Aab and relative link between BPC.

The target of R&R Aab are conserved intracellular polymeric structures composed IMPDH, which formation is induced by IMPDH inhibitors/glutamine deprivation condition.

Representing a distinct class of subcellular structures, R&R are not associated with any known organelles (13). The R&R pattern is a very rare finding of ANA testing in the clinical laboratory, for two reasons:

First of all, R&R pattern is related to some HEp2 cell cultures (14). 

Second, this prevalence is reported to be between 5% and 20% of the HCV infected patients cohort (6,15). 

IMPDH2 is a component of the structures recognized by the R&R Aab (6) it has been related to the HCV treatment with ribavirin (retroviral agent), that is an inhibitor of this enzyme. 

*In vitro* studies showed the ability of ribavirin to induce the intracellular structures that may lead to an anti-R&R response (6,15). Carcamo *et al*. supposed that the induction of Aab may be enhanced by the concomitant administration of a-interferon (6).

However, our attention focused on a hypothetical patient’s cohort/ cases series or case report regarding R&R Aab in absence of HCV infection or antiviral treatment. Keppeke and colleagues reported the presence of one patient with hepatitis B in a cohort of 57 HCV-R&R positive cases (7). Another study performing by Probst and colleagues, using IMPDH2 as a substrate in a radio-immunoprecipitation assay, showed the presence of R&R Aab in non-HCV patient’s cohort, particularly in individuals with concomitant antibodies to actin (14). 

In addition to these evidences, Shaikh’s work reported 39 R&R Aab positive subjects in which only one is HCV-positive patient (16). Pharmacological treatments are note to evoke immunological responses and Aab formation. 

Literature reported one patient under mycophenolate mofetil (MMF) treatment as example of inhibitor to the IMPDH2 enzymatic activity (17). In this case, MMF may cause changes in the molecular structure of IMPDH2 similar to those caused by ribavirin (18). 

However, this hypothesis cannot conciliate with the idea of immunosuppressive action, because these agents would theoretically prevent antibody synthesis. 

Chan *et al*. reported three case patients treated with methotrexate (MTX) that, although it does inhibit purine throughout IMPDH2, but on dihydrofolate reductase (19).

Finally, it is very important to establish if the Literature reported ALD cases with R&R Aab presence. Climent *et al*. reported eight patients with R&R Aab and concomitant AD and they hypotized that these Aab may be a result of autoimmune responses (20). However, Climent and collegues specified that the R&R pattern was not significantly associated with presence of with PBC or AIH (20). 

The same authors performed liver biopsy in 54 R&R-positive patients. These biopsies revealed a high degree of hepatic fibrosis in 38 patients, but not specific histological pattern referred to PBC, AIH and other overlapping conditions (20).

Our work conversely specified the contemporary presence of AMA-M2 and R&R Aab, PCB related symptom and PCB related histological pattern. 

While AMA can be regarded as the most specific autoantibodies in the field of autoimmunity it is quite frustrating that as many as 15% of patients with PBC (11). Patients lacking detectable AMA Aab, especially when IIF is used but otherwise presenting signs and symptoms of PBC should be regarded as affected by “AMA-negative PBC” as they appear to follow a similar natural history when compared to their AMA-positive PCB (11). Immunological biomarkers other than AMAs are useful for this group of patients. These include various ANA detected by IIF. Two distinct patterns are strongly associated with PBC: the multiple nuclear dot (MND) and rim-like/membranous patterns. The target antigens MND patterns are the sp100 protein and the PML protein. The rim-like pattern was associated to antibodies targeting the nuclear pore complex antigen gp210, the nucleoporin antigen p62 and the lamin B receptor (21).

Our work demonstrated that R&R Aab can be present in PBC case. The presence of R&R Aab in our PBC case introduced the possible use of these Aab as a new biomarker in AMA-negative PBC. 

Our results described above reproduce the methodological approach for a complete characterization of complex antibodies profile. In these overlapping situations it is particularly important that routinely used techniques are combined in a correct way, in order to provide a successful characterization of the fine features of particular cases (22-23-24).

The study provided an opportunity to discuss the role of traditional approach by IIF investigation. 

As a part of diagnostic protocols, IIF for detection the ANA remains the non-discussed screening assay in routine. An important question is whether the IIF screening test has sufficient sensitivity to detect all clinically relevant Aab. This deficiency has led some laboratories to leave the IFI as a first step of the diagnostic tool. The ability to identify multiple Aab in a single serum is now made possible by using multiplexed diagnostic platforms. Not only some of these antibodies appear quite similar by routine IIF, but also they are often seen in association with other antibodies and in these cases, the contribution of other technologies can be useful. Finally, these Aab will be considered as ‘‘diagnostic’’ and not as ‘‘esoteric’’ antibodies.

*The future of R&R Aab*

This paper opens the door to the alternative scenario such as the association of these Aab to ALD. To solve this issue, other studies has needed.

In fact a strong, but not exclusive, association does exist between hepatitis C infection and R&R Aab. But Literature review indicated that the R&R pattern cannot be considered only a mere side effect of the antiviral treatment. It may also be a consequence of other factors such as alterations in immune regulation. Our case should be considered a touchable example of this situation: R&R Aab in patients without any clinical/laboratory signs or symptoms of viral Hepatitis or pharmacological therapy. I clearly demonstrate that R&R Aab are not exclusively hallmark of HCV- ribavirin treated patients, but the expression of a complex and partially unknown pathway. 

I would track the possible use of R&R Aab as biomarker of AMA-negative PCB, or so good underline the R&R Aab presence in autoimmune disease.

## Conflict of interests

The authors declare that they have no conflict of interest.
